# Exploring the importance of diversified physical activities in early childhood for later motor competence and physical activity level: a seven-year longitudinal study

**DOI:** 10.1186/s12889-021-11343-1

**Published:** 2021-08-02

**Authors:** Paulina S. Melby, Peter Elsborg, Glen Nielsen, Rodrigo A. Lima, Peter Bentsen, Lars B. Andersen

**Affiliations:** 1 Department of Nutrition, Exercise and Sports, University of Copenhagen, Copenhagen, Denmark; 2 Health Promotion, Steno Diabetes Center Copenhagen, The Capital Region of Denmark, Gentofte, 2820 Danmark; 3 Center for Clinical Research and Prevention, Copenhagen University Hospital, Bispebjerg and Frederiksberg, 2000 Frederiksberg, Denmark; 4 Research, Innovation and Teaching Unit, Parc Sanitari Sant Joan de Déu, Sant Boi de Llobregat, Barcelona, Spain; 5 Department of Geosciences and Natural Resource Management, University of Copenhagen, 1958 Frederiksberg C, Denmark; 6 Department of Sport, Food and Natural Sciences, Faculty of Education, Arts and Sports, Western Norway University of Applied Sciences Campus Sogndal, Bergen, 5020 Norway; 7 Faculty of Education, Arts and Sports, Western Norway University of Applied Sciences, Bergen, 5020 Norway

**Keywords:** Adolescent, Childhood, CoSCIS, Diversified physical activity, Leisure time, Motor skills, MVPA, Physical literacy, Recreational activities, SEM

## Abstract

**Background:**

Research indicates that childhood motor competence (MC) can predict physical activity (PA) levels later in life and it has been argued that frequently engaging in a wide diversity of physical activities will eventually improve children’s MC. However, no longitudinal or experimental studies have confirmed this theoretical rationale. The aims of this study are to explore the longitudinal associations between diversified physical activities at age six and later MC and PA (time spent in moderate-to-vigorous PA) (age nine and 13). Furthermore, we explore to what extent the longitudinal association between diversified physical activity and PA is mediated by MC.

**Methods:**

Longitudinal data from the Copenhagen School Intervention Study were used for this analysis, where 704 participated (69% response rate). Diversified physical activity (self-reported), MC (The *Körperkoordinationstest für Kinder* battery of postural stability and locomotor skills) and PA (accelerometer) were assessed in the children at age six, age nine and age 13. A total of 654 participated in at least two of the measures and, therefore, were included in the analysis. Two structural equation models were constructed, with diversified physical activity at age six and MC and PA at age nine as predictors of PA and MC at age 13.

**Results:**

The data from both models demonstrated good model fit. Diversified physical activity at 6 years of age was significantly associated with physical activity and MC at age 13, when adjusting for sex, age, intervention, weight, height, and previous levels of PA and MC. Diversified physical activity at age six was also positively associated with PA and MC at age nine, which were, in turn, positively related to PA at age 13 but to a lesser degree than diversified PA at age six. The association between diversified physical activity at age six and PA at age 13 was not mediated by MC at age nine.

**Conclusions:**

The results of this study indicate that diversified physical activity at age six is important for the development of MC and PA in adolescence. Increasing the diversity of children’s daily physical activities, not only the amount and intensity, seems important for future PA behavior and thereby health promotion in a life course perspective.

## Background

Physical activity (PA) is part of a healthy lifestyle as it reduces the risk of developing cardiovascular diseases and other non-communicable diseases later in life [1]. Current recommendations state that children and adolescents should participate in at least 60 min of moderate-to-vigorous physical activity (MVPA) per day [2]. Globally, 78% of adolescent boys and 84% of adolescent girls (age 11–17) did not meet this recommendation in 2010 [3], in addition to this, PA levels for both sexes decrease with age [4]. Further, the transition from childhood to adolescence is associated with a decline in PA [5]. There have been reports suggesting that PA levels peak around age 12 [6] and then decline as individuals age [7–9]. It is essential to explore factors that determine participation in PA among adolescents in order to increase their PA. One understudied aspect to consider is the participation in different physical activities through childhood which may improve the ability to participate in a more broad range of activities later in life through improved motor competence (MC) and more specific skills, but also through experiences in different types of PA, including gained knowledge and understanding (e.g. on how to engage in different activities and settings) and through increased confidence and motivation towards PA [10, 11]. To reduce terminology inconsistencies and align with other research in the field, the terminology ‘motor competence’ is used in this paper as a global term to describe goal-directed human movement [12, 13].

MC have been shown to be related to PA levels and fitness [14–17] and also seems related to academic and cognitive performance in children [18, 19]. MC are defined as;” *… an organized series of basic movements that involve the combination of movement patterns of two or more body segments*.” [[15];^p52^] and are often classified into three components: postural stability (e.g., static and dynamic balance), object control (e.g., catching and throwing) and locomotor movements (e.g., running and jumping). It is argued that postural stability is crucial to the development of the two other components of MC, i.e., locomotor movements and object control [20, 21]. MC are, considered as ‘building blocks’ for the development of more complex and sport-specific movement skills. Studies have found that the most ideal period to learn these skills is during preschool and early school years [21, 22]. It is suggested that underdeveloped MC potentially creates a barrier for participation in PAs [22, 23].

Some researchers suggest a reciprocal relationship between MC and PA that will strengthen over time [14, 22, 23]. A positive relationship between MC and PA in childhood and youth has been strongly asserted in cross-sectional studies [15, 16, 24]. However, whether it is a high level of PA that improves MC or better MC that makes children participate more in PA is more uncertain due to a lack of longitudinal studies. Some longitudinal studies indicate that childhood MC can predict later PA [14, 25, 26], while a recent systematic review found no consistent evidence for PA as a predictor of MC [17]. Additionally, a positive relationship between MC and physical fitness in childhood and has been asserted [12, 14, 27], and there are some evidence that the association between MC and PA is mediated by cardiorespiratory fitness [14, 23, 28] . Presuming that MC in childhood is a crucial factor impacting future PA levels, it is important to investigate what determines the development of MC.

Physical literacy is an individual’s disposition to participate in and maintain PAs throughout the life course [10]. Whitehead spearheaded the concept and defined physical literacy as: “… the motivation, confidence, physical competence, knowledge and understanding to value and take responsibility for engagement in physical activities for life” [10;^pp13^]. Other definitions focus on PL as consisting of different, yet somewhat interdependent, domains. Often an affective, physical and cognitive domain are described [29] which cover concepts such as confidence, motivation, physical capacity (i.e. fitness and strength), MC, knowledge, and understanding.

The attention towards the multidimensional construct of physical literacy and e.g. its potential role for population health and wellbeing has increased in recent years [29–32]. This increased interest in physical literacy stands on a belief that the interaction of the domains brings additional explanatory value beyond the sum of the concepts included in the construct [30].

Dudley et al. [33] argued that physical literacy promotes MC, which in turn is related to PA [15, 16, 24] and physical fitness [12, 14]. A large scale Canadian study on physical literacy has shown it to be associated to variables such as sedentary behavior, cardiorespiratory fitness, adherence to PA guidelines and weight status in children aged 8 to 13 [34]. In the theory of physical literacy, it is suggested that all body movements will have an impact on the development of MC, and the young child should accordingly engage in many diverse range of movement activities to create the best circumstances for developing good MC [10]. This is supported by research on the consequences of early specialization in sports, which shows that early specialization in one sport and activity-specific skills, can result in limited range of MCs [35], which may result in decreased participation in PA in adulthood [36] and dropout from organized PA [37]. Further, the holistic theory of physical literacy offers an approach where future engagement in PA throughout the life is affected by not only physical capacity (i.e. MC), but also knowledge and understanding (e.g. on how to engage in different activities and settings), and confidence and motivation for different PA’s. Our theoretical rationale for this explorative investigation is that engaging in a diverse range of physical activities at an early age is impacting later PA levels through other mechanisms than solely MC.

**Fig. 1:**
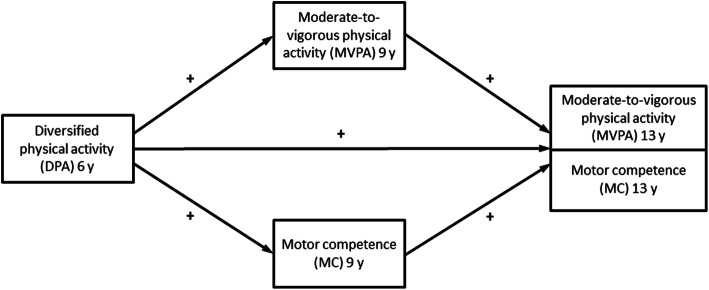
Structural equation models

Therefore, the aims of this longitudinal study are to investigate the longitudinal relationship between diversified physical activities, MC and MVPA (see Fig. 1) with an emphasis on the impact of leisure-time diversified physical activities in early childhood (age six) on the development of later MC and MVPA (age nine and age 13). An aim is also to explore to what extent the longitudinal association between diversified physical activities and MVPA can be explained (is mediated) by MC at age nine. Our theoretical rationale is that diversified physical activities in childhood can increase later MC and PA in children and that the longitudinal association between diversified physical activities at age six and MVPA at age 13 is partly mediated by MC at age nine.

Figure 1 shows the two theorized models, with moderate-to-vigorous physical activity (MVPA) at age 13 (model 1) and motor competence (MC) at age 13 (model 2) as the outcome respectively.

## Materials and methods

### Study design and setting

The participants of this study were from intervention and control schools of the CoSCIS-study (The Copenhagen School Child Intervention Study) [38], which was designed as a school-based PA intervention study. Because the complete methodology has been previously published [38], the following methods section only describes the variables used in the analysis presented in this paper. In brief, the study began in 2001, and data were collected when the children were attending 0th grade (mean age 6.3 years), 3rd grade (mean age 9.5 years) and 7th grade (mean age 13.3 years). Forty-six 0th graders from 18 schools located in two comparable suburban municipalities in terms of socioeconomic status and population size – Ballerup and Tårnby – in the Copenhagen area participated in the study. Classes in the schools from Ballerup were given two additional lessons of PE from 0th grade until 3rd grade. There was no increase in PA levels in 3rd grade [39], nor did the intervention show an effect on MC [38]. In this paper, the design is analyzed as a longitudinal observational cohort study.

### Sampling and participants

Of the 1024 children from the 18 schools, 704 participated in the study (69% response rate) at baseline. These children were representative in height and weight of the whole population of children in the two municipalities [40].

A total of 991 children participated in the study for at least one of the measurement years. Of these, 654 of the participants (52% boys) gave consent and contributed data in at least two of the measures and, therefore, were included in the analysis. Table 1 presents the number of participants and the percentage of the total sample (*N* = 654) participating in the tests each year.

**Table 1:** Participants (N) in each measure and percentage of total sample

	***6 years***		***9 years***		***13 years***		***All ages***	
	***N***	***sample%***	***N***	***sample%***	***N***	***sample%***	***N***	***sample%***
MC	601	92%	601	92%	429	66%	387	59%
MVPA	499	76%	454	69%	327	50%	218	33%
MC & MVPA	456	70%	441	67%	316	48%	190	29%
DPA	515	79%						
All measures	364	56%					169	26%

*MC* motor competence, raw-score from KTK battery, *MVPA* moderate-to-vigorous physical activity, accelerometer data for at least 3 days, *DPA* diversified physical activity, index based on questionnaire

An analysis of missing data (see Table 2) indicates that the samples are similar in all factors except age and MC. Participants with missing data in at least one measure scored significantly lower in the *Körperkoordinationstest für Kinder* (KTK) battery in 0th grade (4.8% lower) and in 3rd grade (4.8% lower). There were no differences between dropouts regarding the amount of MVPA or diversified physical activities.

**Table 2:** Comparison of missing data for two samples

	***Sample with DPA 6y***				***Sample with all timepoints***			
	***N***		Mean (SD)		***N***		Mean (SD)	
	Valid	Missing	Valid	Missing	Valid	Missing	Valid	Missing
Sex (boy)	515	137	51.5%	54.7%	169	483	52.7%	52.0%
Int. (I-group)	515	137	59.2%	52.6%	169	483	63.9%	55.7%
Age (baseline)	481	129	6.28 (0.35)	6.36 (0.36)*	168	442	6.29 (0.34)	6.30 (0.36)
W/H 6y	478	121	0.21 (0.03)	0.21 (0.03)	159	440	0.21 (0.03)	0.21 (0.03)
W/H 9y	487	123	0.24 (0.04)	0.24 (0.04)	169	483	0.24 (0.03)	0.24 (0.04)
W/H 13y	367	74	0.31 (0.05)	0.31 (0.04)	169	483	0.31 (0.05)	0.31 (0.05)
DPA 6y					169	346	8.57 (3.30)	8.58 (3.41)
MVPA 6y	398	100	76.00 (26.84)	78.57 (22.67)	169	329	77.49 (25.85)	76.02 (26.18)
MVPA 9y	364	88	72.90 (23.85)	72.29 (23.75)	169	283	73.93 (25.53)	72.09 (22.74)
MVPA 13y	278	47	54.80 (22.23)	54.73 (25.88)	169	156	54.11 (20.53)	55.52 (24.98)
MC 6y	474	127	121.05 (26.99)	116.10 (27.50)	169	432	124.28 (25.73)	118.33 (27.53)*
MC 9y	481	120	196.24 (34.22)	190.73 (36.46)	169	432	202.33 (31.45)	192.33 (35.56)*
MC 13y	357	72	249.82 (29.34)	246.69 (29.87)	169	260	251.48 (28.73)	247.87 (29.83)

Comparison of missing data (mean and standard deviation) for sample with DPA measure and sample with all measures at all timepoints. *p-value < 0.05; *Int.* intervention/control-group; *W/H* weight/height ratio; *MVPA* moderate-to-vigorous physical activity (min/day); *MC* motor competence (raw score from KTK-battery); *DPA* diversified physical activity; 6y = 6 years of age; 9y = 9 years of age; 13y = 13 years of age

### Ethics approval and consent to participate

In Denmark, only biomedical research and research projects that entail a risk for participants can receive a Trial Registration Number through ethics review by a Regional Ethics Board. Written information about the study was given to all school principals, teachers and parents before the start of the study, and informed consent were obtained from the legal guardians of all participants. The children were informed about the different parts of the measurements they participated in. It was made clear that they did not have to participate in any of the measurement activities they did not want to and that they could drop out anytime regardless of having initially accepted to participate.

The study obtained approval from the local ethics committee of the University of Copenhagen (reference KA00011gm).

### Data collection

#### Measurement of amount of diversified physical activity

Drawing on the approach and theories of physical literacy [10], we created a variable to measure and assess the influence of diversity of the children’s daily PAs. As no validated instrument for measuring specific activities was available at the time of the data collection (baseline measure was carried out in 2001), such as e.g. CLASS [41] and APARQ [42], a measure was developed by a cross disciplinary research group. As children’s leisure time activities are age and culture specific the questionnaire was developed to fit the age group (6 years) and the Danish context.

In 0th grade, children completed a 1 week recall questionnaire of leisure and sport habits together with a parent. The last week were probed in an effort to minimalize recall bias, and the assistance of the parent were encouraged to minimize issues with understanding of the questions. In the questionnaire, numerous leisure activities were listed (a total of 28 activities) and were checked off if the child had engaged in the activity outside of school and sports-clubs during the past week.

Ten of the leisure activities involved PA defined as involving body movement as central to the activity (playing with a ball; bicycling; skating/ice skating; playing a game of tag; playing hopscotch/jumping rope; using scooters; going to the skating rink, swimming pool or playground; and dancing). Furthermore, the child’s participation in different club-organized sports was recorded with a maximum score of four different sports. Based on both the leisure-time self-organized PA’s (amount of different activities per week, min/max 0–10, mean 3.1) and club organized sports activities (number of different sports per week, min/max 0–4, mean 0.79) the number of different activities the child had participated in was calculated.

To take into account that not only the number of different activities completed but also the time spent engaging in them influences the development of physical literacy, the weekly number of leisure-time sports training sessions (min/max 0–4, mean 0.9) and the weekly number of hours playing outdoors during leisure time (min/max 0.5–7, mean 4.2) were added.

This total score of the number of different activities added to the number of activity sessions was labeled *diversified physical activity*. A high score in this variable reflects that the child has engaged in many different kinds of PAs for a considerable amount of time which, according to the concept of physical literacy, should give a good condition for developing MC, but also knowledge of different ways of being active, and feeling confident with different PA’s. The minimum and maximum value of the four components added up to measure total amount of diversified physical activities can be seen in Table 3.

**Table 3:** Sample size, min/max, mean and standard deviations

		Possible range		Descriptive for sample			
	N	min	max	min	max	mean	SD
Age (baseline)	610			5.46	7.69	6.3	0.35
W/H 6y	599			0.15	0.4	0.21	0.03
W/H 9y	610			0.16	0.44	0.24	0.04
W/H 13y	441			0.21	0.47	0.31	0.05
DPA 6y	515	0.5	31	0.5	18	8.57	3.37
Outdoor play (times/week)		0.5	7	0.5	7	4.2	2.18
Sport (training sessions/week)		0	7	0	4	0.9	0.88
Leisure-time self-organized PA (amount of different activities/week)		0	10	0	10	3.1	2.23
Number of different sports		0	7	0	4	0.79	0.852
MVPA 6y (min/day)	498			25.94	169.38	76.52	26.05
MVPA 9y (min/day)	452			20.5	143.22	72.78	23.81
MVPA 13y (min/day)	325			9.28	140.17	54.79	22.75
MC 6y (KTK score)	601			39	204	120	27.15
MC 9y (KTK score)	601			89	291	195.14	34.72
MC 13y (KTK score)	429			140	327	249.29	29.42

*p-value < 0.05; *W/H* weight/height ratio; *DPA* diversified physical activity; *MVPA* moderate-to-vigorous physical activity (min/day); *MC* motor competence (raw score from KTK-battery); 6 y = 6 years of age; 9 y = 9 years of age; 13 y = 13 years of age

The measure of diversified physical activities has been used for other purposes [39].

#### Measurement of physical activity (PA)

The MTI 7164 activity monitor (Actigraph, Fort Walton Beach, Florida, USA) was used to measure the daily amount of MVPA in the children at baseline, and the Actigraph GT1M was used at the follow-ups in 3rd and 7th grade. The monitors have been validated in children in several studies [43]. A 10-s epoch was chosen since short bursts of activity are characteristic of children’s PA [44]. Due to the restricted memory of the MTI 7164 monitors, the recording period was limited to 4 days.

For the measurement period, we selected two school days and two weekend days. The monitor was worn by the children on an elastic belt, and to allow for familiarization, it was worn for 1 day before recording. The data were cleaned for non-wear (≥ 30 min of consecutives zero counts) to distinguish between periods of sedentary behavior and periods where the monitor was not worn. Data were included in the final dataset if the monitor collected activity data for at least 3 days with a minimum of 10 h of valid recordings per day (0th grade: 4 days *n* = 322, 3 days *n* = 204; 3rd grade: 4 days *n* = 298, 3 days *n* = 156). The children having only 3 days of valid days had only one weekend-day. In 7th grade, the monitor was worn for up to 7 days, but the same inclusion criteria were used (7th grade 6–7 days *n* = 233, 3–5 days *n* = 92). Average wear time and wear days for 0th grade was 42.24 (±11.77 SD) hours and 3.33 (±0.84 SD) days and for 3rd grade it was 44.35 (±12.14 SD) hours and 3.37 (±0.81 SD). Average wear time in 7th grade was 76.86 h (±31.94 SD) and 5.67 (±2.16 SD) days. For each time point MVPA measurement were collected on the same months (September, October, November, February, March and April). Each school (and hence each child) in the study were measured on the same month of the year at each time point.

Data were analyzed for minutes per day (7 am-11 pm on the included days) spent performing moderate-to-vigorous physical activity (MVPA). We used Evenson cut-points [45], as they have been validated for the age groups participating in this study [46]. The threshold for MVPA was set at ≥2298 counts per minute, which reflects approximately four metabolic equivalents (METs) and medium exertion (e.g., walking 5.2 km/h) and above [46].

#### Measurement of motor competence (MC)

The German standardized test battery the *Körperkoordinationstest für Kinder* (KTK) [Body coordination test for children] [47] was chosen to assess MC. The KTK battery was developed to examine gross body coordination in children. The subtest tasks are very different from the daily activities of children and sports-specific skills, and the outcome reflects the children’s MC within the two aspects of motor competence: postural stability and locomotor skills [47]. The KTK battery is suitable for children within the age range of 5 to 15 years, and is useful for longitudinal research [48–51], and has been used for talent detection and identification purposes [52, 53]. The KTK battery has shown good test-retest reliability (*r* = 0.80–0.96) as well as good inter-test reliability between the items (*r* = 0.60–0.80) [47, 54].

The test was administered by the research team and was carried out in a classroom or sports hall, where the children rotated between test stations in groups of four to five. The test consisted of four subtasks: 1) balancing backwards on three beams with widths decreasing from six to three centimeters (accumulated score from three trials for each beam, with a maximum score of 72 points), 2) hopping on one foot over foam blocks with increasing height (maximum of 78 points for both legs), 3) jumping sideways with legs together (score was the total number of successful jumps performed over two 15 s trials), and 4) moving sideways using two wooden platforms (score was the total number of transitions performed over two 20s trials) [47]. The scores for each sub-task were summed into a raw motor quotient.

### Statistical approach

Descriptive statistics and bivariate correlations were calculated in SPSS 22.0 (IBM Corp, Armonk, NY, US). Longitudinal associations between diversified physical activities, MC and MVPA were investigated through structural equation modeling in the R package lavaan [55]. Missing values were estimated based on full information maximum likelihood estimation. By using this method, it was not possible to adjust for the cluster effect (by school and classes nested within school). Although it would not be possible to account for the nesting structure of the data, we decided to estimate the missing values to avoid selection bias, which would have a larger impact on the quality of the analysis and results compared to the variance not accounted for by the nesting structure of the data [56]. The variance within the cluster (school) for MC at age 13 was ICC = 0.076 (95%CI = 0.026;0.194) and for MVPA at age 13 ICC = 0.035 (95%CI = − 0.006;0.133). The distributions of study variables were inspected visually and were considered to be normally distributed. The following criteria for a good model fit were used: chi-square statistics (chi-square/df < 5.00), comparative fit index (CFI > 0.95), Tucker-Lewis index (TLI > 0.95), and root mean square error of approximation (RMSEA < 0.06) [57].

Longitudinal associations of diversified physical activities at age six, MC at age nine and MVPA at age nine with MVPA at age 13 were investigated, as well as longitudinal associations of diversified physical activities at age six, MC at age nine and MVPA at age nine with MC at age 13 (see theorized models in Fig. 1). Adjustments were made for sex, age, extra PE intervention/municipality, weight/height ratio, MVPA and MC at age six (see control paths in Tables 5 and 6). Covariation between all exogenous variables was allowed. Significance tests were 2-tailed and *P*-values below 0.05 were considered statistically significant.

Since we controlled for sex and age in the analysis, we chose to use the raw KTK score instead of the age- and gender-specific motor quotient.

## Results

### Study variables

Mean age of the participants were at baseline were 6.3 years, 52% of the participants in the sample were boys and 57,8% of the sample attended an intervention school. Minimum, maximum, mean scores and standard deviations for all variables are reported in Table 3.

Intercorrelations for the variables are reported in Table 4. As seen, MC had a strong correlation over time (*r* = 0.5–0.7). There was no significant correlation of diversified physical activities with MVPA at age six (*r* = .1). MVPA at age nine did not correlate with MC at age nine or MC at age 13.

**Table 4:** Variable inter-correlations (pearson’s R)

		1	2	3	4	5	6	7	8	9
***6 y***	**1**: W/H									
	**2**: MVPA (min/day)	.00								
	**3**: MC (KTK score)	−.17**	.18**							
	**4**: DPA	.07	.10	.11*						
***9 y***	**5**: W/H	.89**	−.01	−.22**	.04					
	**6**: MVPA (min/day)	.07	.32**	.05	.12*	.05				
	**7**: MC (KTK score)	−.30**	.17**	.70**	.13*	−.38**	.02			
***13 y***	**8**. W/H	.74**	−.06	−.10*	.04	.85**	.02	−.27**		
	**9**: MVPA (min/day)	−.09	.33**	.11	.24**	−.05	.34**	.13*	−.08	
	**10**: MC (KTK score)	−.23**	.15*	.50**	.17*	−.33**	.05	.63**	−.35**	.20**

**p-value < 0.001; *p-value < 0.05; *W/H* weight/height ratio; *MVPA* moderate-to-vigorous physical activity (min/day); *MC* motor competence (raw score from KTK-battery); *DPA* diversified physical activity; 6 y = 6 years of age; 9 y = 9 years of age; 13 y = 13 years of age

### Longitudinal associations among diversified physical activities, MVPA and MC

**Fig. 2:**
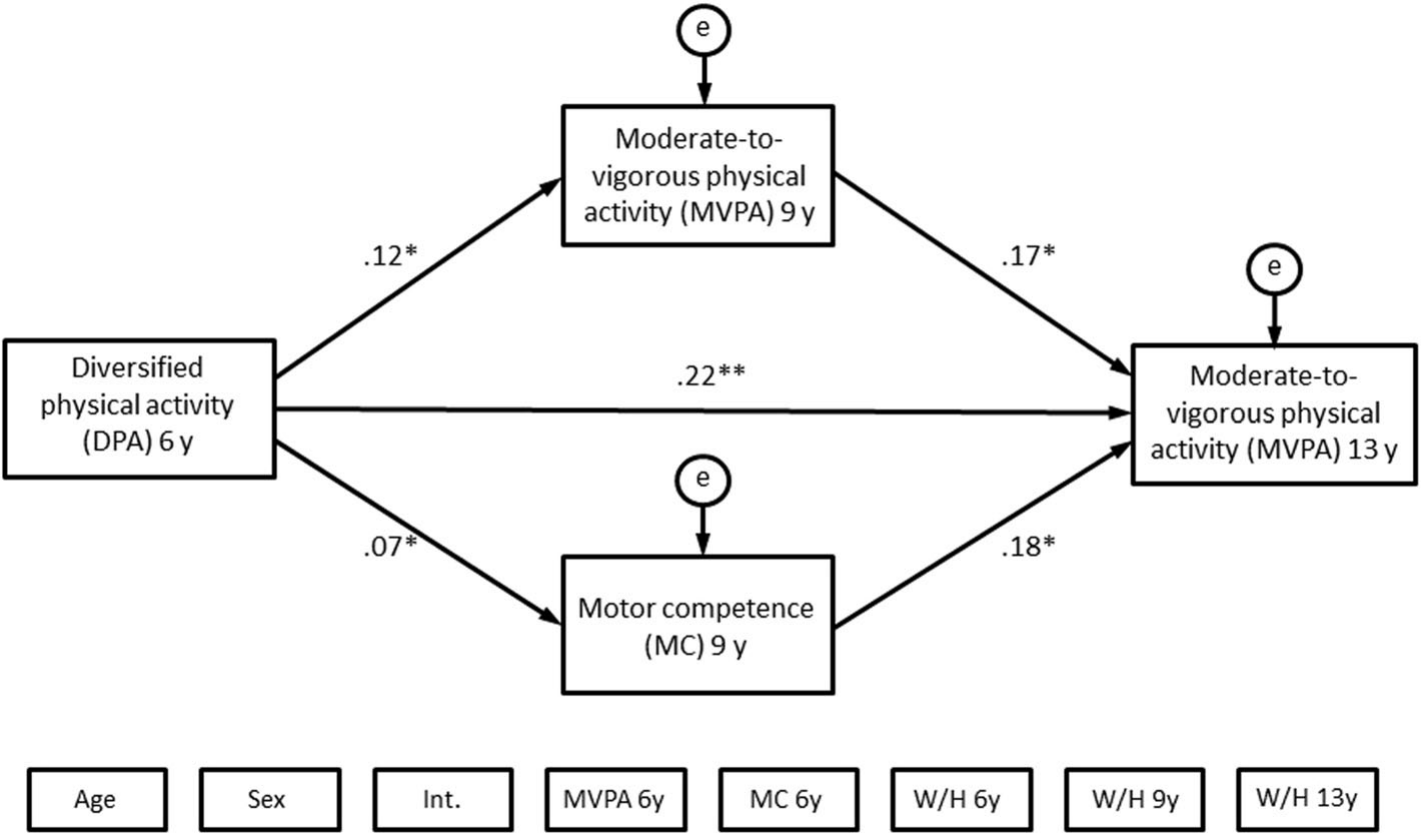
Path coefficients of the structural equation model with MVPA at age 13 as outcome

**Fig. 3:**
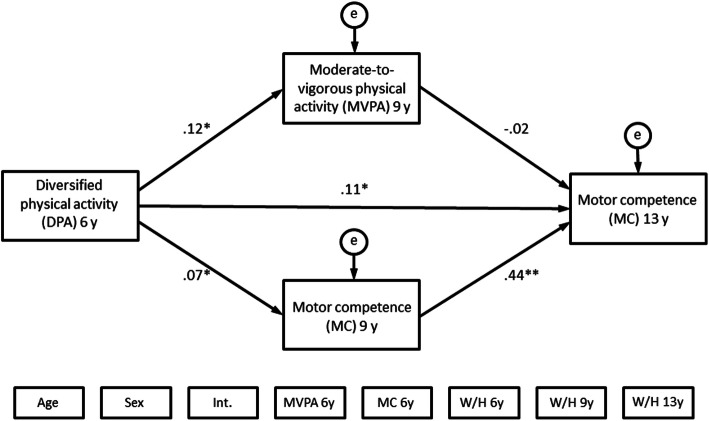
Path coefficients of the structural equation model with MC at age 13 as outcome

Figures 2 and 3 show the final structural equation models with MVPA at year 13 and MC at year 13 respectively as the outcomes. Note that all covariate variables in Figs. 2 and 3 are presented without arrows to improve the visual clarity of the model. The models were run while controlling for confounding variables.

Figure 2 shows path coefficients of the final structural equation model with moderate-to-vigorous physical activity (MVPA) at 13 years as the outcome. All the parameters (β) were standardized and were statistically significant. Covariate variables are placed below the model. Covariation between all exogenous variables was allowed. See control paths in Table 5. DPA = diversified physical activities; MVPA = moderate-to-vigorous physical activity; MC = motor competence; Int. = intervention/control-group; W/H = weight / height ratio; 6 y = 6 years of age; 9 y = 9 years of age; 13 y = 13 years of age.

**Table 5:** Information on paths in the structural equation model with MVPA at age 13 as outcome

***From***		***To***	***B***	***SE***	***Std B***	***p***
**DPA 6 y**	**—>**	**MC 9 y (KTK score)**	**0.71**	**0.33**	**.07**	**.03**
**DPA 6 y**	**—>**	**MVPA 9 y (min/day)**	**0.87**	**0.36**	**.12**	**.02**
**DPA 6 y**	**—>**	**MVPA 13 y (min/day)**	**1.45**	**0.36**	**.22**	**<.01**
**MC 9 y**	**—>**	**MVPA 13 y (min/day)**	**0.12**	**0.05**	**.18**	**.02**
**MVPA 9 y**	**—>**	**MVPA 13 y (min/day)**	**0.16**	**0.06**	**.17**	**<.01**
***Control Paths***						
MVPA 6 y	—>	MC 9 y (KTK score)	0.07	0.04	.05	.12
**MVPA 6 y**	**—>**	**MVPA 9 y (min/day)**	**0.27**	**0.05**	**.30**	**<.01**
**MVPA 6 y**	**—>**	**MVPA 13 y (min/day)**	**0.18**	**0.05**	**.21**	**<.01**
**MC 6 y**	**—>**	**MC 9 y (KTK score)**	**0.83**	**0.04**	**.65**	**<.01**
MC 6 y	—>	MVPA 9 y (min/day)	−0.02	0.04	−.02	.72
MC 6 y	—>	MVPA 13 y (min/day)	−0.10	0.06	−.12	.11
Sex*	—>	MC 9 y (KTK score)	2.62	1.99	.04	.19
Sex*	**—>**	**MVPA 9 y (min/day)**	**−7.33**	**2.19**	**−.15**	**<.01**
Sex*	**—>**	**MVPA 13 y (min/day)**	**−11.66**	**2.28**	**−.26**	**<.01**
Age (baseline)	—>	MC 9 y (KTK score)	−5.75	3.00	−.06	.06
Age (baseline)	—>	MVPA 9 y (min/day)	2.83	3.34	.04	.40
Age (baseline)	—>	MVPA 13 y (min/day)	2.54	3.58	.04	.48
Int.	—>	MC 9 y (KTK score)	−0.21	2.01	−.01	.92
Int.	—>	MVPA 9 y (min/day)	−1.59	2.23	−.03	.48
Int.	—>	MVPA 13 y (min/day)	0.88	2.21	.02	.70
W/H 6 y	—>	MC 9 y (KTK score)	123.64	76.44	.10	.12
W/H 6 y	—>	MVPA 9 y (min/day)	24.36	91.13	.03	.79
**W/H 6 y**	**—>**	**MVPA 13 y (min/day)**	**− 223.52**	**86.49**	**−.29**	**.01**
**W/H 9 y**	**—>**	**MC 9 y (KTK score)**	**− 288.91**	**58.04**	**−.32**	**<.01**
W/H 9 y	—>	MVPA 9 y (min/day)	−5.67	68.38	−.01	.93
**W/H 9 y**	**—>**	**MVPA 13 y (min/day)**	**200.17**	**86.28**	**.34**	**.02**
W/H 13 y	—>	MVPA 13 y (min/day)	−62.77	42.44	−.15	.14
***Mediating Paths***						
DPA 6 y — > MVPA 9 y — > MVPA 13 y			0.14	0.07	.02	.06
DPA 6 y — > MC 9 y — > MVPA 13 y			0.08	0.05	.01	.10

Path coefficients (*β*), standard error (*SE*), standardized regression weights (*Std B*), and *p*-values for paths in the structural equation model with moderate-to-vigorous physical activity (MVPA) at age 13 as the outcome. Bold text indicates significant paths (*p*-value > 0.05). *Boy is coded 1, girl is coded 2. *DPA* diversified physical activities; *MVPA* moderate-to-vigorous physical activity; *MC* motor competence; *Int*. intervention (coded 1)/control-group (coded 2); *W/H* weight / height ratio; 6 y = 6 years of age; 9 y = 9 years of age; 13 y = 13 years of age

The structural equation model analysis revealed that diversified physical activities at age six and MVPA and MC at age nine were longitudinally associated with MVPA at age 13 (Fig. 2). All standardized path coefficients (β) and *p*-values are presented in Table 5. MC at age nine (β = 0.18, *p* = .02) and MVPA at age nine (β = 0.17, *p* < .01) were associated with MVPA at age 13 (Fig. 2, Table 5). MC at age six was neither associated with MVPA at age nine nor with MVPA at age 13 (Table 5, control paths).

Diversified physical activities at age six was associated with MVPA at age 13 (β = 0.22; *p* < .01). Diversified physical activities at age six was also associated with MVPA (β = 0.12, *p* = .02) and MC (β = 0.07, *p* = .03) at age nine. The association between diversified physical activities at age six and MVPA at age 13, was not significantly mediated by MC at age nine (β = 0.08, *p* = 0.10) or MVPA at age 9 (β = 0.14, *p* = .06). The model adequately fitted data (CFI = .987, TLI = .919, RMSEA = .067) [57].

Figure 3 shows path coefficients of the final structural equation model with motor competence (MC) at year 13 as the outcome. All the parameters (β) were standardized, and all variables except for MVPA at age nine were statistically significant. Covariate variables are placed below the model. Covariation between all exogenous variables was allowed. See regression weights for control paths in Table 6. DPA = diversified physical activities; MVPA = moderate-to-vigorous physical activity; MC = motor competence; Int. = intervention/control-group; W/H = weight / height ratio; 6 y = 6 years of age; 9 y = 9 years of age; 13 y = 13 years of age.

**Table 6:** Information on paths in the structural equation model with MC at age 13 as outcome

***From***		***To***	***B***	***SE***	***Std B***	***p***
**DPA 6 y**	**—>**	**MC 9 y (KTK score)**	**0.69**	**0.33**	**.07**	**.04**
**DPA 6 y**	**—>**	**MVPA 9 y (min/day)**	**0.86**	**0.36**	**.12**	**.02**
**DPA 6 y**	**—>**	**MC 13 y (KTK score)**	**1.05**	**0.36**	**.11**	**<.01**
**MC 9 y**	**—>**	**MC 13 y (KTK score)**	**0.39**	**0.05**	**.44**	**<.01**
MVPA 9 y	—>	MC 13 y (KTK score)	−0.03	0.06	−.02	.62
***Control Paths***						
MVPA 6 y	—>	MC 9 y (KTK score)	0.06	0.04	.05	.14
**MVPA 6 y**	**—>**	**MVPA 9 y (min/day)**	**0.27**	**0.05**	**.30**	**<.01**
MVPA 6 y	—>	MC 13 y (KTK score)	0.03	0.05	.03	.50
**MC 6 y**	**—>**	**MC 9 y (KTK score)**	**0.83**	**0.04**	**.66**	**<.01**
MC 6 y	—>	MVPA 9 y (min/day)	−0.02	0.04	−.02	.67
**MC 6 y**	**—>**	**MC 13 y (KTK score)**	**0.23**	**0.06**	**.23**	**<.01**
Sex*	—>	MC 9 y (KTK score)	2.6	1.98	.04	.19
Sex*	**—>**	**MVPA 9 y (min/day)**	**−7.12**	**2.20**	**−.15**	**<.01**
Sex*	**—>**	**MC 13 y (KTK score)**	**−4.75**	**2.17**	**−.08**	**.03**
Age (baseline)	—>	MC 9 y (KTK score)	−5.46	3.01	−.06	.07
Age (baseline)	—>	MVPA 9 y (min/day)	2.63	3.34	.04	.43
**Age (baseline)**	**—>**	**MC 13 y (KTK score)**	**−6.59**	**3.39**	**−.07**	**.05**
Int.	—>	MC 9 y (KTK score)	−0.03	2.01	.00	.99
Int.	—>	MVPA 9 y (min/day)	−1.78	2.23	−.04	.43
Int.	—>	MC 13 y (KTK score)	0.41	2.16	.01	.85
W/H 6 y	—>	MC 9 y (KTK score)	118.04	76.41	.10	.12
W/H 6 y	—>	MVPA 9 y (min/day)	39.86	91.48	.05	.66
**W/H 6 y**	**—>**	**MC 13 y (KTK score)**	**264.50**	**86.96**	**.25**	**<.01**
**W/H 9 y**	**—>**	**MC 9 y (KTK score)**	**− 284.67**	**57.98**	**−.31**	**<.01**
W/H 9 y	—>	MVPA 9 y (min/day)	−16.48	68.65	−.03	.81
**W/H 9 y**	**—>**	**MC 13 y (KTK score)**	**−164.86**	**83.27**	**−.20**	**.05**
**W/H 13 y**	**—>**	**MC 13 y (KTK score)**	**− 139.06**	**40.52**	**−.24**	**<.01**
**Mediating Paths**						
DPA 6 y — > MVPA 9 y — > MC 13 y			−0.03	0.05	−.01	.63
**DPA 6 y — > MC 9 y — > MC 13 y**			**0.27**	**0.13**	**.04**	**.04**

Path coefficients (*β*), standard error (*SE*), standardized regression weights (*Std B*), and *p*-values for paths in the structural equation model with motor competence (*MC*) at age 13 as the outcome. Bold text indicates significant paths (*p*-value > 0.05 *Boy is coded 1, girl is coded 2. *DPA* diversified physical activities; *MVPA* moderate-to-vigorous physical activity; MC = motor competence; Int. = intervention (coded 1)/control-group (coded 2); *W/H* weight / height ratio; 6 y = 6 years of age; 9 y = 9 years of age; 13 y = 13 years of age

Figure 3 shows the structural equation model analysis in which MC at the age of 13 years was the outcome and Table 6 presents all standardized path coefficients (β) and *p*-values. MVPA at age nine was not associated with MC at age 13 (Table 6). Nor did we find an association between MVPA at age six and MC at age nine or between MVPA at age six and MC at age 13 (Table 6, control paths).

Like in the first model, diversified physical activities at age six was associated with MVPA at age nine (β = 0.12, *p* = .02) and MC at age nine (β = 0.07, *p* = .04). Diversified physical activities at age six was also associated with MC at age 13 (β = .11; *p* < .01), and the association was partly mediated by MC at age nine (β = .04; *p* = .04). We observed a strong direct association between MC at age nine and MC at age 13 (β = .44; *p* < .01). The model adequately fitted the data (CFI = 1.00, TLI = 1.01, RMSEA = .058) [57].

## Discussion

The results of this study indicate that having a diverse physical activity pattern in early childhood is associated with higher levels of objectively measured PA 7 years later.

Diversified physical activities at age six was positively associated with MVPA and MC at age nine. MVPA and MC at age nine were also positively related to MVPA at age 13 but to a lesser degree than diversified physical activities at age six. Furthermore, the association between diversified physical activities at age six and MVPA at age 13 was not significantly mediated by MC or MVPA at age 9 and there were no longitudinal associations from MC at age six to MVPA at age nine or at age 13.

Although not a high beta value, considering that the association between diversified physical activities at age six and MVPA at age 13 was observed over a period of 7 years, the β-value of 0.22 is noteworthy. To the best of our knowledge, no studies have explored the longitudinal association between diversified physical activities and MVPA. From our theoretical rationale based on the concept of physical literacy [10], diversified physical activities at a young age is important for later levels of MVPA. The observed associations between early diversified physical activities and later PA levels could be an indication that early diversified physical activities results in inspiration to choose more widely when deciding how to be physically active later in life, or/and that skills more specific to the physical activities available to and undertaken by children and youth in their everyday life than perhaps general motor skills might be important to overall PA. A diverse pattern of PAs would most likely lead to skills, knowledge and understanding for a varied and broad number of activities and coordination patterns that an individual can recall on when needed and in diverse PA context and settings. Further, the non-significant mediation of MC could be an indication that diversified physical activity in early childhood impacts later levels of PA through other mechanisms than solely MC, like e.g. knowledge and understanding (e.g. on how to engage in different activities and settings), and confidence and motivation for different PA’s. Also, the work of Hulteen and colleagues argues that various elements affects the development of fundamental movement skills and engagement in PA across the lifespan [58]. Their conceptual model shares some of the same mechanisms as the theory of physical literacy. Future studies, e.g., with an experimental intervention design, should investigate the association between childhood diversified physical activities and PA and the underlying mechanisms.

The association between diversified physical activities at age six and MVPA at age 13 was not significantly mediated by MC. This is quite surprising as it has been theoretically argued that diversified physical activities contributes to well-developed MC, which then enable participation in many PAs [6]. The non-significant mediation could be due to that the assessment of MC was rather narrow (only postural and locomotor skills).

Diversified physical activities at age six was associated with MC at age nine and 13. We did not find any longitudinal associations between MVPA (at age six or at age nine) and MC at an older age. As described in the introduction, there is no clear evidence for MVPA as a predictor of MC [17]. Results from our study have expanded on this sparse knowledge, showing that diversified physical activities at age six is important for MC at a later age, even when controlling for MVPA. The results indicate that MC does not develop solely by taking part in high amounts of MVPA, but by engaging in many different types of activities at an early age. It has been suggested that MC needs to be taught, practiced and reinforced, especially later in childhood [12]. Interventions targeting MC development in PE curriculum has shown to be effective [59], but the enduring effect on MC or PA after intervention has not been demonstrated. While acknowledging the impact of teaching MC through an instructional approach, diversified physical activities may be an effective, accessible and sustainable way to develop MC in early childhood years.

To our knowledge four other studies have investigated the longitudinal associations between MC and PA [14, 25, 60, 61] in samples similar to our sample (between approximately 4–10 years at baseline). Generally, it is difficult to compare results from studies investigating MC and PA, as many different measurement batteries are used to assess MC and self-reported measures of PA are still common. A meta-analysis concluded that correlates of MC differ according to the operationalization of MC [17]. An explanation to why our results are contradictory to other studies that find relationship between MC and PA, could be that the KTK battery used in our study does not assess manipulative skills. Barnett and colleagues [60] showed in a study of 276 school children (mean age of 10 years) that manipulative skills were associated with self-reported PA 6 years later It might be that this aspect of fundamental movement skills is a better predictor for e.g. adolescent PA, since these type of skills (e.g., kicking, catching, throwing), are required in many traditional organized sports. Thus, because the KTK battery used in our study does not assess manipulative skills, comparisons should be considered with caution. Two other longitudinal studies of the association between MC at an early age (4–6 years) and later levels of PA are not consistent. Lopes and colleagues [25] assessed MC in 285 children (mean age of 6 years) using the KTK battery, and PA was assessed (self-reported) over four consecutive years. The authors observed that children in the highest MC tertile at baseline showed the smallest drop in levels of PA up to 4 years later compared to the middle and low MC tertiles. This result indicates that MC at a young age is important for later PA. In a study of 207 children, McKenzie and colleagues [61] assessed MC at ages four, five and six and self-reported PA at age 12. They observed no association between childhood MC and PA at age 12 and discussed the limitations of the battery used for assessing MC, as it consisted of three single tests (lateral jump, catch a ball, and balance on one foot). In our study, we also did not find MC at age six to be associated with PA at age 13, while however MC at age nine was.

### Strengths and limitations

The results of this study should be interpreted with caution knowing that, the diversified physical activities measure is not previously validated or extensively studied. This is especially the case since some of the results of this study contradict previous studies. However, on the other hand, the preliminary results of this study also illustrate the necessity of future studies developing and applying validated measures of diversified physical activities and investigates its importance for life long PA participation.

To our knowledge, this is the first study to explore the relations between diversity of childhood physical activities and longitudinal development of MC and MVPA. The longitudinal design of seven years with two follow-ups and the objective measurement of PA improve the credibility of the results. Furthermore, the participants were followed during childhood and early adolescence, when foundations of PA behaviors are formed that seem to continue until adulthood [62]. Even though the study has an observational design, the associations observed are highly robust to dropout. Even though the data used are relatively old, the associations have general validity, as it is unlikely that the relationship between diversified physical activities in childhood and MVPA in adolescence would have changed since the data was collected in 2008.

Although accelerometers, compared to self-reported measurements, are considered a valid instrument for assessing PA in children and youth, some limitations are associated with this method. First, the accelerometers were not worn during swimming. Most of the intervention schools included swimming in the extra PE lessons; thus, this extra activity, due to the method of measurement, was not recorded. Our findings of a weak association between MC and PA levels should be viewed in the light of that, due to the memory capacity of the accelerometers, they only gave a three- to four-day snapshot measure of the children’s daily PA at age six and nine and these results contradict what has been found in a number of review on the topic [15–17]. Additionally, the accelerometers did not measure participation in cycling, which contributes a substantial amount of PA in this population of Danish children.

Another limitation in this study is that we did not control for the cluster effect. The decision not to adjust for the cluster effect was made in order to use structural equation modeling. Structural equation modelling has been shown to be beneficial in investigating longitudinal associations while avoiding selection bias by using full information maximum likelihood estimation to impute missing values. Thus, the structural equation model analysis should be noted as a strength of the study despite the aforementioned limitation. Although our analysis indicates that the sample with full data is similar to the sample with missing data, an additional limitation was the amount of missing data in the primary indicators and outcomes.

The complexity of the model and number of parameters lead us to the decision of not having any latent variables in the structural equation model [56]. Future research could investigate and develop the items and measurement model behind the diversified physical activities measure in more depth.

Furthermore, another limitation of the study is that we did not measure diversified physical activities at 9 years of age, and perhaps larger β-values could have been observed from diversified physical activities at age nine for MVPA and MC at age 13. It would also be possible to explore if cross-sectional results differ in comparison to longitudinal findings. It may be, that diversified physical activities is only possible for individuals when MC are well developed. Future research should investigate whether MC and/or MVPA at age six is associated with diversified physical activities at an older age. Future research should look into the interrelationship among all the variables from a more explorative perspective. Furthermore, future research could also explore whether the associations are different for boys and girls.

Additionally, we advocate that future research develop and adopt a validated measurement tool for diversified physical activities. A suggestion for measuring diversified physical activities in children may possibly be via ecological momentary assessment [63, 64] over the course of a week. Although being a subjectively instrument, the ecological momentary assessment holds several advantages. With this instrument it would be possible to explore the variety of activities, durations and settings were the child’s PA is undertaken during a week. To date, an objectively method to measure diversity and context of PA is still to be developed.

### Implications for practice

These results propose a new dimension for PA-related health recommendations for children, which currently only highlight the importance of being physically active at a moderate-to-vigorous intensity. While the health benefits of PA are well-documented [65], the long term effect of interventions aiming to increase PA amongst children are at best moderate. Focusing interventions on promoting diversified physical activities may have greater potential, as it addresses the underlying domains of physical literacy that has been theoretical argued to be important for supporting PA levels during the whole life course [10].

Several studies show that the amount of daily MVPA is associated with several health outcomes in young children [2], but the optimal amount of MVPA for children and young people is a debated topic. However, if diversified physical activities at age six increases the likelihood of being physically active in adolescence and maybe even later in life, then more focus should be placed on encouraging children to engage in a diverse range of PAs. Viewed through a life course perspective [66], our results indicate that investments in helping children engage in a wide variety of PAs is more important for later PA than solely increasing the time spent in MVPA in childhood.

Interventions targeting diversified physical activities in early childhood should be conducted to test its potential for maintenance in PA behavior across childhood and adolescent years. Some countries, Canada in particular, have already incorporated the importance of diversified physical activity as an important aim of their physical education curriculums [67]. Our study supports this focus for physical education in schools and perhaps even for kindergarten activities. In addition, agents involved in leisure-time PAs for children, such as sports clubs and associations, should consider the importance of providing diversified PAs rather than focusing on a single sport. The debate and studies regarding early specialization vs. diversification have mostly centered on whether it is more beneficial for later performance levels to engage in a diverse range of activities (i.e., diversified physical activities) instead of focusing on one specific sport during childhood [68]. The results of this study show that diversification also appears to be beneficial for general activity levels later in life. Another structural aspect of the importance of children’s daily PA is the environment in which the child lives and interacts, e.g., the number of play facilities available, which seems to be important for activity levels [69]. It seems likely that the diversity of play facility types available is one aspect that can enable diversified physical activities in childhood.

## Conclusion

This study indicates that diversified physical activity in early childhood is associated with higher levels of objectively measured physical activity 7 years later in adolescence. Diversified physical activity at age six is also positively associated with physical activity and motor competence at age nine, which are, in turn, positively related to physical activity at age 13 but to a lesser degree than diversified physical activity at age six. This indicates that increasing the diversity of children’s daily physical activities, not only the amount and intensity of physical activity, is an important aim for lifelong physical activity participation and thereby health promotion.

## Data Availability

The datasets generated and analysed during the current study are not publicly available because we did not acquire consent from our participants or their parents to share data outside our research group. A desensitized dataset are available from the corresponding author on reasonable request.

## Abbreviations

CoSCIS: The Copenhagen school intervention study
DPA: Diversified physical activity
KTK: Körperkoordinationstest für kinder (body coordination test for children)
MC: Motor competence
MVPA: Moderate-to-vigorous physical activity
PA: Physical activity
